# Correction: Directionality theory and mortality patterns across the primate lineage

**DOI:** 10.1007/s10522-024-10155-1

**Published:** 2024-11-15

**Authors:** Lloyd A. Demetrius, Anand Sahasranaman, Martin Ziehe

**Affiliations:** 1 Dept. of Organismic and Evolutionary Biology, Harvard University, Cambridge, Mass 02138 USA; 2 Centre for Complexity Science, Imperial College London, London, SW72AZ UK; 3 Faculty of Forest Genetics and Forest Ecology, University of Gottingen, Busgenweg 2, 37077 Gottingen, Germany

**Correction: Biogerontology** 10.1007/s10522-024-10134-6

In this article the wrong figure appeared as Fig. 6; the figure should have appeared as shown below.

**Fig. 6:**
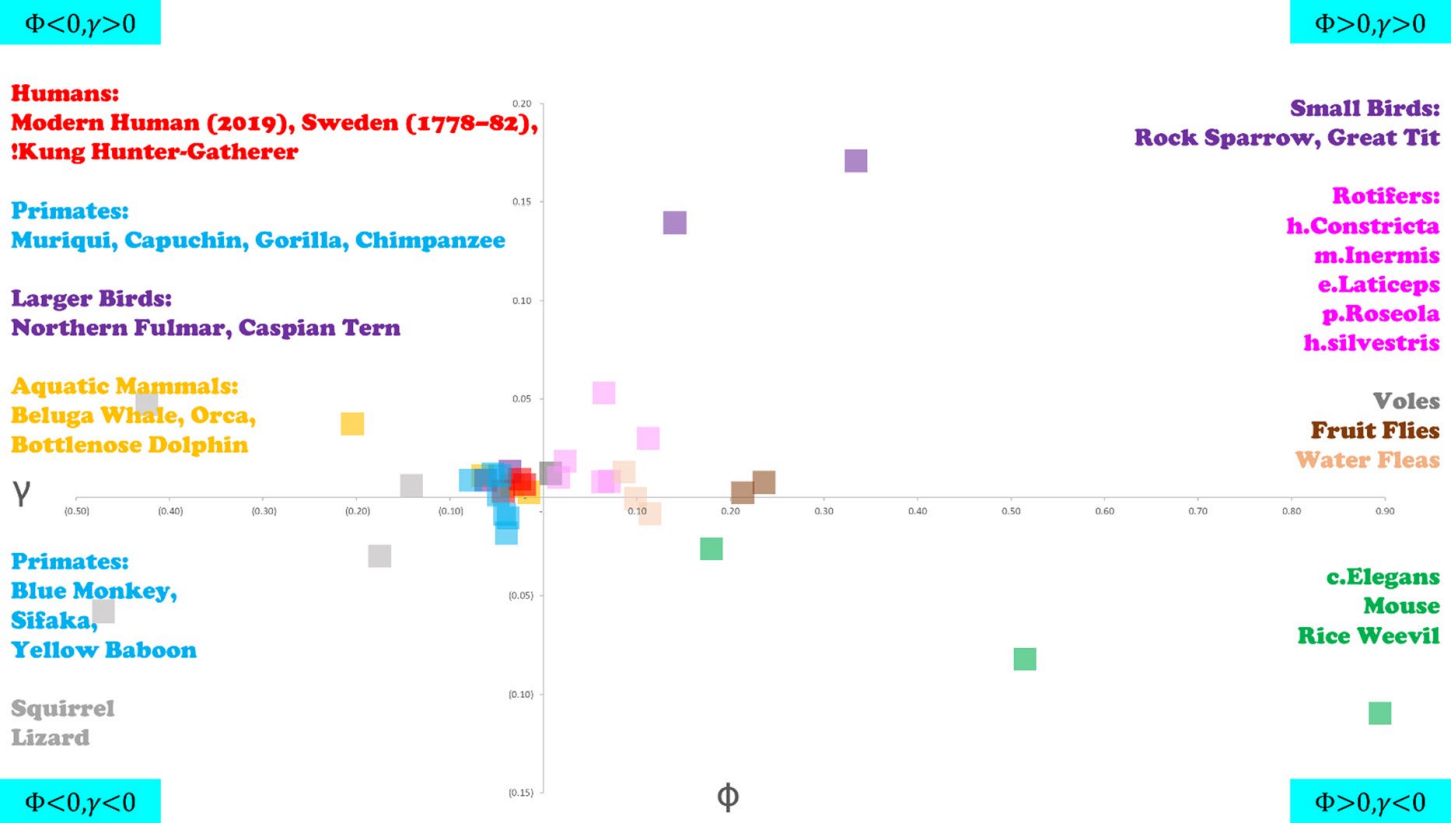
Distribution of species on the Φ – *γ* plane

The original article has been corrected.

